# Consumption of caffeine-containing beverages improves sustained attention and alertness but has no effect on strength or static muscular endurance

**DOI:** 10.1017/jns.2026.10122

**Published:** 2026-08-03

**Authors:** Lia Jiannine, Catherine Holen, Tobin A. Silver, Cassandra Evans, Jose Antonio

**Affiliations:** 1 Nova Southeastern University, USA; 2 Dept. of Health and Human Performance, Nova Southeastern University, USA

**Keywords:** Attention, Energy drinks, Reaction time, Supplement, Vigilance

## Abstract

Energy drinks are widely consumed for their caffeine content and related ergogenic effects. Many energy drinks also contain additional ingredients purported to enhance both physical and cognitive performance; however, it remains unclear whether these benefits are attributable solely to caffeine or the synergistic effect of other ingredients. The purpose of this study was to assess the effects of an energy drink vs. a caffeine-matched control on measures of sustained attention. Using a randomised, double-masked design (*n* = 116), subjects were assigned to an energy drink (C4 – Nutrabolt®) or a caffeine-matched control (200 mg caffeine). The following assessments were conducted before and approximately 45 min after consuming an energy drink or caffeine-matched control: psychomotor vigilance test (PVT), MemTrax (memory), handgrip strength, and wall-sit endurance. Both the energy drink and the caffeine-matched control improved pre vs. post for the PVT (i.e. reaction time improved); however, there were no differences in the delta score. The energy drink group showed a significant decrease in the MemTrax assessment (% correct), whereas reaction time increased, with no change in the control group. However, there were no differences in the delta score between groups for either the % correct score or reaction time. There were no significant differences in the handgrip or wall-sit assessments between the two groups. There are no significant differences between the effects of consuming an energy drink or caffeine. Both beverages improved sustained attention and alertness likely due to the effects of caffeine. There were no differences in memory, handgrip strength, or wall-sit endurance.

## Introduction

Caffeine is among the most commonly consumed psychoactive substances and is a key component in commercial energy drinks.^(1)^ Its ergogenic effects on exercise performance have been extensively researched vis-à-vis endurance, strength, and mental performance.^(2–5)^ While some research has shown improvements in reaction time, attention, and vigilance, other studies have reported no significant effects.^(3)^ Similarly, findings on the influence of energy drinks on muscular strength and endurance have been inconsistent.^(6–8)^ Although some studies suggest energy drinks may offer benefits beyond those of caffeine alone, others indicate no notable differences compared to placebo or caffeine-only drinks.^(9)^ Results from various studies likely differ due to caffeine doses, experimental protocols, and measures. Energy drinks may contain additional ingredients beyond caffeine (200 mg), including vitamin B_12_, beta-alanine (BA), and betaine. BA is a non-proteogenic amino acid that serves as a precursor to carnosine that buffers hydrogen ions in skeletal muscle. It improves anaerobic performance by delaying fatigue and enhancing the ability to sustain high-intensity exercise, particularly in efforts lasting longer than 60 s.^(10)^ Betaine is a trimethylglycine naturally occurring in foods such as spinach and beets. Literature suggests it improves performance by increasing intracellular creatine levels.^(11)^ Cobalamin, commonly known as vitamin B_12_, has many functions in the body. It is thought to confer an ergogenic aid due to its role in the production of red blood cells and serving as a cofactor for energy production and red blood cell production.^(12)^ However, the efficacy of these added ingredients combined with caffeine is unclear and warrants additional studies. Thus, the purpose of this study was to evaluate the effects of a commercially available energy drink on tasks related to mental and physical performance – such as sustained attention (i.e. psychomotor vigilance test (PVT)), memory (i.e. MemTrax), lower body muscular endurance (i.e. wall-sit), and handgrip strength – relative to a positive control drink containing an equivalent amount of caffeine.

## Methods

### Overview

**Figure 1.**
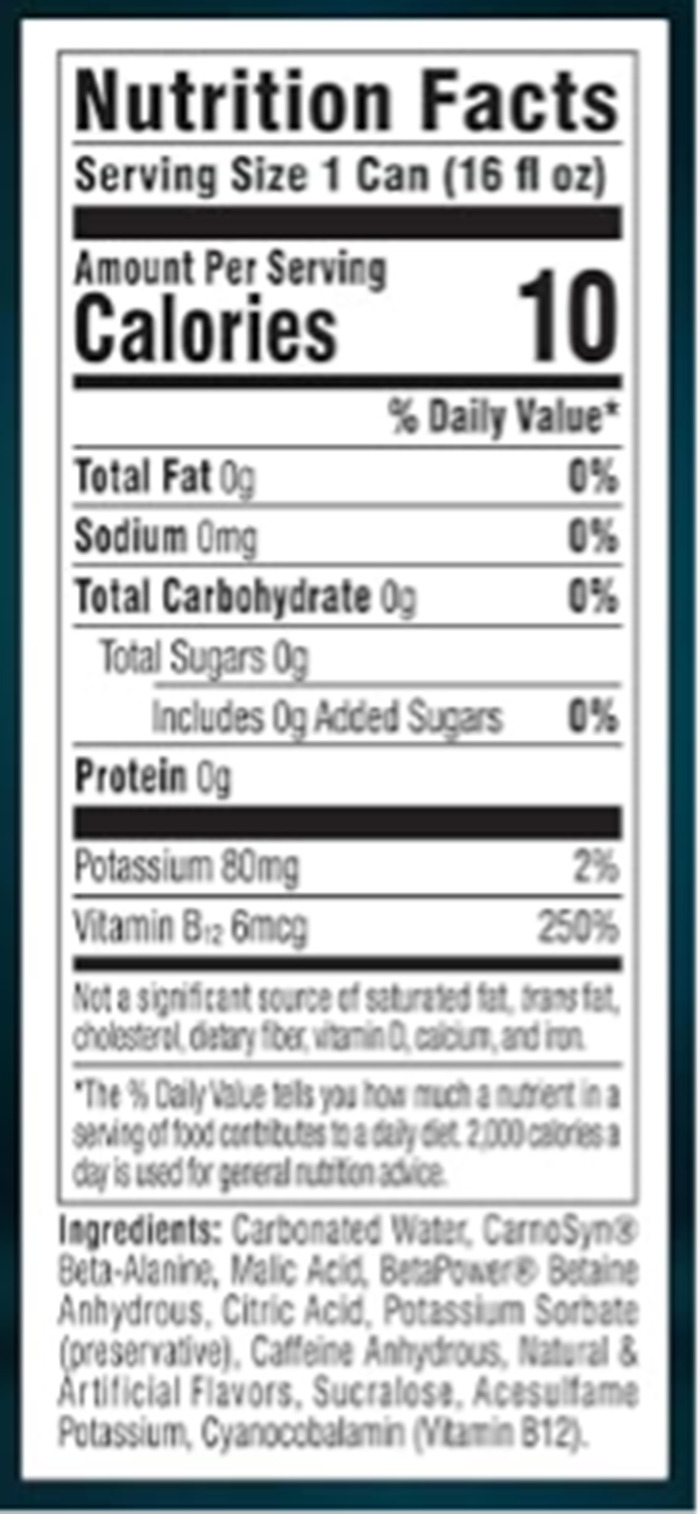
Nutrition facts panel of C4 energy drink.

**Figure 2.**
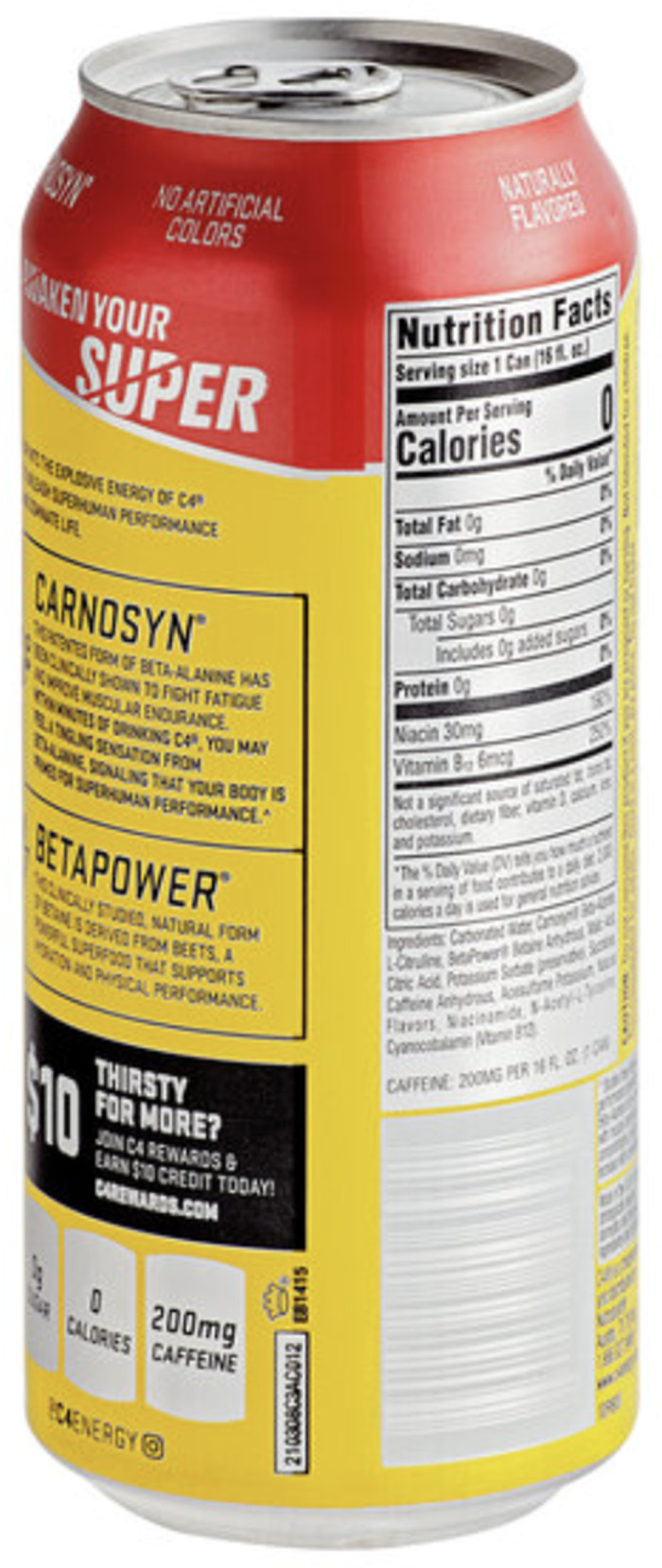
Caffeine content of C4 energy drink.

This study used a two-arm, randomised, double-blind design. Inclusion criteria were as follows: adults aged 18 years or older who engaged in physical activity at least three times per week for a minimum of 30 min per session. Individuals with diabetes or pre-existing heart or kidney disease, as determined by self-report, were excluded from participation. Participants were randomly assigned to consume either a commercially available energy drink (C4 – Nutrabolt®) containing 200 mg of caffeine (Figures 1 and 2) or a caffeine-matched control beverage (200 mg caffeine mixed with Crystal Light). All procedures involving human subjects were conducted in accordance with the Declaration of Helsinki and were approved by the university’s Institutional Review Board (IRB# 2025-32).

Participants were instructed to arrive at the lab fasted, abstaining from vigorous exercise and caffeine intake. Exercise history was evaluated using a standardised questionnaire, and additional information regarding habitual caffeine intake and exercise behaviours was obtained through onsite interviews conducted in the laboratory. Body composition was assessed via bioelectrical impedance analysis (InBody 270). Participants completed a battery of tests, including the PVT for sustained attention, MemTrax for memory performance, handgrip strength, and wall-sit endurance. Next, participants consumed the assigned beverage and waited 30 min before completing a repeated battery of tests.

### Participants

A total of 116 participants completed the study. Participant characteristics are presented in Table 1.

**Table 1.** Physical characteristics means ± SD Table 1 long description.

Characteristics	Treatment *N* = 60 (18 males)	Control *N* = 56 (19 males)	*p*-Value
Age, years	21 ± 3	20 ± 1	0.4083
Height, cm	169 ± 11	171 ± 10	0.4155
Body mass, kg	69 ± 16	75 ± 29	0.2021
Body fat, %	23 ± 9	23 ± 9	0.7457
Aerobic exercise per week, minutes	239 ± 268	198 ± 244	0.4024

There were no significant differences between the groups within the body of Table 1.

### Psychomotor vigilance test

The PVT is a widely used cognitive assessment tool that measures sustained attention and reaction time. It involves responding to a visual stimulus (i.e. a number) that appears at random intervals by tapping the electronic device (e.g. an iPad) as quickly as possible. The test records reaction times and false starts. The test is performed on a computer tablet. We have used this assessment previously.^(9)^ Participants were tested before consuming the drink (treatment or control) and then retested ∼45 min post-consumption. This was repeated for all assessments, except for the wall sit.

### MemTrax memory test

The MemTrax test is an online continuous recognition memory assessment designed to evaluate episodic memory performance, attention, and reaction time. The test is brief, typically lasting approximately 2 min. The key metrics derived from this test include the percentage correct of identified repeated images, as well as the response time.^(13)^

### Handgrip strength test

The handgrip strength test assesses the strength of the finger flexors. Using a hand dynamometer (Jamar Hydraulic Hand Dynamometer 5030J1), the test required each participant to squeeze the device with maximum effort while seated with the elbow at a 90-degree angle and the forearm in a neutral position. The best of three attempts was recorded. The dominant hand was tested.

### Wall-sit endurance test

The wall-sit test assessed lower-body muscular endurance, targeting the muscles of the lower extremities. During the test, participants placed their backs flat against a wall with their knees bent at a 90-degree angle, their thighs parallel to the ground, their feet flat, and their feet shoulder-width apart. The individual maintains this position for as long as possible without hand support or shifting posture. Subjects were not allowed to see the timer. Time was recorded once the subject could no longer hold the position or experienced significant form breakdown. Participants were tested one time ∼45 min after consuming the drink (treatment or control).

### Statistics

All statistical analyses were performed using GraphPad Prism (GraphPad Software, San Diego, CA, USA). All data are expressed as the mean and standard deviation. A paired *t*-test was used to determine if within-group changes were significant (*p* < 0.05). An unpaired *t*-test was used to compare the delta score between the groups. The study used convenience sampling.

## Results

**Figure 3.**
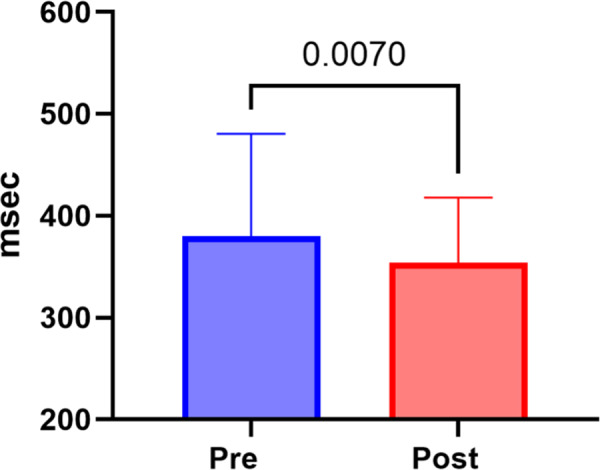
Pre- vs. post-assessment of the PVT for the control drink. Legend: All data are expressed as the mean and SD. There was a significant decrease in the reaction time pre to post for the caffeine-matched control group.

**Figure 4.**
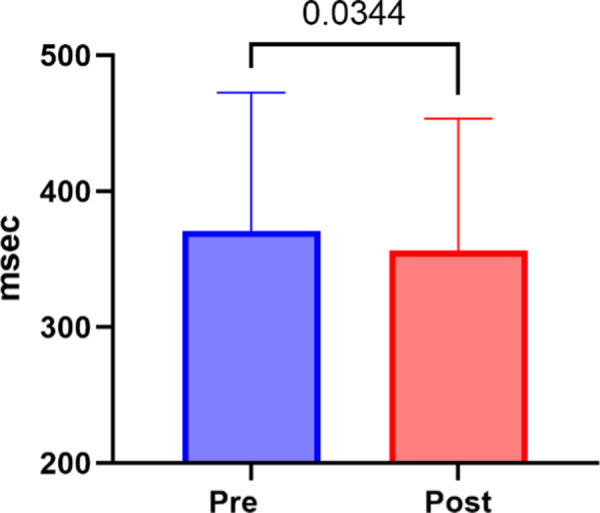
Pre vs. post-assessment of the PVT for the energy drink. Legend: All data are expressed as the mean and SD. There was a significant decrease in the reaction time pre to post for the energy drink group.

**Figure 5.**
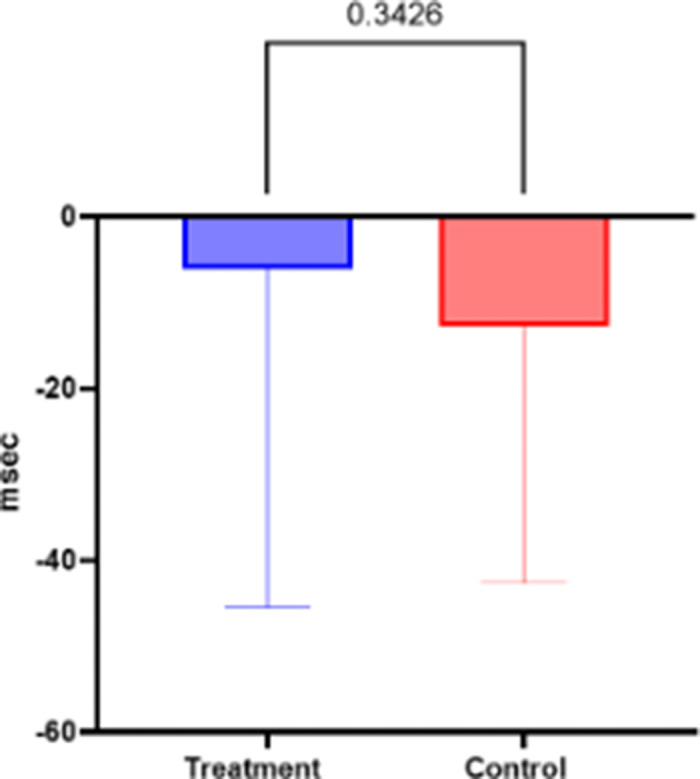
The delta score between groups for the PVT. Legend: All data are expressed as the mean and SD. There were no differences between groups for the PVT delta score.

The physical characteristics of the treatment and control groups are shown in Table 1 and 2. Both the energy drink and control groups demonstrated significant improvements in reaction time on the PVT from pre- to post-testing (*p* = 0.0344 and *p* = 0.0070, respectively; Figures 3 and 4); however, there were no differences in the delta score (Table 3 and Figures 3–5).

**Table 2.** Physical characteristics Table 2 long description.

Characteristics	Treatment *N* = 60 (18 males)	Control *N* = 56 (19 males)	*p*-Value
Age, years	21 ± 3	20 ± 1	0.4083
Height, cm	169 ± 11	171 ± 10	0.4155
Body mass, kg	69 ± 16	75 ± 29	0.2021
Body fat, %	23 ± 9	23 ± 9	0.7457
Aerobic exercise per week, minutes	239 ± 268	198 ± 244	0.4024

Data are the mean and standard deviation (SD). There were no significant differences between the groups.

**Table 3.** Performance metrics Table 3 long description.

Characteristics	Treatment *N* = 60 (18 males)	*p*-Value	Control *N* = 56 (19 males)	*p*-Value
PVT pre (msec)	371 ± 102		380 ± 101	
PVT post (msec)	356 ± 97*	0.0344	354 ± 101*	0.0070
Delta	−15 ± 59		−26 ± 74	0.3426
MemTrax % correct pre	95 ± 5		91 ± 11	
MemTrax % correct post	92 ± 6*	0.307	93 ± 5	0.0614
Delta	−3 ± 5		2 ± 11	0.1596
MemTrax RT pre (sec)	0.77 ± 0.14		0.78 ± 0.18	
MemTrax RT post (sec)	0.79 ± 0.13*	*p*< 0.0001	0.78 ± 0.17	0.3950
Delta	0.02 ± 0.10		0.00 ± 0.12	0.1596
Handgrip pre (kg)	38 ± 13		38 ± 13	
Handgrip post (kg)	39 ± 13	0.0055	38 ± 13	0.2661
Delta	1 ± 4		0.0 ± 0.1	0.5485

Data are the mean and SD. *Significantly different than the pre-test. PVT treatment *p* = 0.0344, PVT control *p* = 0.0070, MemTrax % correct treatment *p* < 0.0001, MemTrax reaction time treatment *p* = 0.307. There were no significant differences for any of the other assessments (pre vs. post), the wall sit, and the delta scores between groups.

The energy drink group showed a significant decrease in accuracy on the MemTrax assessment (% correct; *p* < 0.0001), whereas reaction time increased, with no change in the control group. However, there were no differences in the delta score between groups for either the % correct score or reaction time. Handgrip strength also increased significantly following energy drink consumption (*p* = 0.0055). There were no significant differences in the handgrip or wall sit assessments between the two groups.

No other variables showed statistically significant changes.

## Discussion

This investigation assessed the effects of consuming a commercially available energy drink (C4 – Nutrabolt®) versus a caffeine-matched control on measures of cognitive and physical performance. Although both interventions demonstrated improvements in sustained attention, as assessed by the PVT, no significant differences were observed between the groups. This is in line with a previous study we conducted in which we compared a commercially available energy drink (Gorilla Mind®) with a caffeine-matched control.^(9)^ Similar to the current investigation, Gorilla Mind energy drink also contains 200 mg of caffeine. Using a randomised, counterbalanced, crossover design, participants completed the following baseline assessments: the Profile of Mood States (POMS), PVT, handgrip strength (HG), and 1-min push-ups (PU) before consuming either the energy drink or the caffeine control. Thirty to 45 min post-consumption, the tests were repeated. Following a 1-week washout period, the participants repeated the protocol using the alternate beverage. Although the energy drink group demonstrated improved reaction time on the PVT, the difference in performance (delta score) between the two drinks was not statistically significant (*p* = 0.3391). Similarly, no significant differences were noted for mood (*p* = 0.152), handgrip strength (*p* = 0.499) or push-up performance (*p* = 0.209).^(9)^ This suggests that the additional ingredients in the energy drink, such as taurine, BA, and betaine, did not confer benefits beyond those attributable to caffeine alone.

Interestingly, the energy drink group exhibited a significant decrease in memory performance (% correct) on the Memtrax assessment, coupled with an increase in reaction time (i.e. performance was worse). Conversely, the control group showed no changes. While caffeine has been shown to enhance certain aspects of cognitive function, including attention and alertness, its effects on memory are less consistent. A recent study evaluated whether acute intake of an energy drink (C4S) could enhance cognitive function, gaming performance, and mood in healthy young adult gamers. Results showed that C4S significantly improved several cognitive domains, including cognitive flexibility, executive function, sustained attention, motor speed, psychomotor speed, and working memory. Gaming performance, particularly in the visuospatial task of Tetris, also improved.^(14)^ On the other hand, Red Bull energy drink, which is a combination of caffeine and taurine, did not affect short-term memory.^(15)^ The decline in memory accuracy observed in the energy drink group of the current study may be attributed to factors such as overstimulation or potential interactions between caffeine and other ingredients. Nevertheless, further investigation is warranted to elucidate these mechanisms.

Handgrip strength showed significant improvements, although similar changes were not observed for wall-sit in the caffeinated group. Prior studies have shown that caffeinated energy drinks can significantly increase handgrip strength. For instance, in resistance-trained men, the ingestion of a caffeinated energy drink improved isometric handgrip strength in both hands compared to a placebo.^(16)^ Similarly, elite junior tennis players experienced a ∼4.2% increase in handgrip force after consuming a caffeinated energy drink.^(17)^ However, these investigations compared a caffeinated energy drink to a placebo. The current investigation used a positive control with an equivalent amount of caffeine.

It should be noted that the study population consisted of young, healthy adults and cannot necessarily be generalised to other populations (e.g. older adults, teenagers, etc.). In addition, the time frame of testing (i.e. 45 min post-consumption) may not capture the peak effects of the drink. It may be worthwhile to examine a longer time frame (e.g. 60–90 min post-consumption). Nonetheless, the results of the current investigation suggest that consuming 200 mg of caffeine, whether as part of an energy drink or as a singular ingredient, effectively enhances sustained attention but does not significantly impact memory or physical performance under the parameters of this investigation. Future research should consider the following: higher doses or alternative formulations of added ingredients, different populations (e.g. athletes and older adults) and additional cognitive and physical performance metrics. It should be noted that the dose of caffeine used in this investigation was approximately 2.8 mg per kilogram body mass (i.e. based on the mean body mass of the research participants). Caffeine enhances performance during exercise when consumed at doses between 3 and 6 mg per kg of body mass. While the minimum effective dose is still uncertain, evidence suggests it could be as low as 2 mg/kg.^(2)^ Nonetheless, a dose of 3 mg/kg or greater is likely necessary to induce more potent ergogenic effects.

## Conclusion

No improvements between conditions were observed; however, both groups experienced significant improvements in alertness and sustained attention. There were no effects on memory, handgrip strength, or wall-sit endurance. This suggests that consuming 200 mg of caffeine, whether it is part of an energy drink or a stand-alone ingredient, induces significant improvements in sustained attention and alertness.

## Table 1 Long description

A table comparing physical characteristics of treatment and control groups. The table has 6 rows and 5 columns. The columns are labeled Characteristics, Treatment, Control, and p-Value. The Treatment column has data for 60 participants (18 males), and the Control column has data for 56 participants (19 males). The rows are labeled Age (years), Height (cm), Body mass (kg), Body fat (percent), and Aerobic exercise per week (minutes). Row 1: Age, years: Treatment 21 ± 3, Control 20 ± 1, p-Value 0.4083. Row 2: Height, cm: Treatment 169 ± 11, Control 171 ± 10, p-Value 0.4155. Row 3: Body mass, kg: Treatment 69 ± 16, Control 75 ± 29, p-Value 0.2021. Row 4: Body fat, percent: Treatment 23 ± 9, Control 23 ± 9, p-Value 0.7457. Row 5: Aerobic exercise per week, minutes: Treatment 239 ± 268, Control 198 ± 244, p-Value 0.4024.

Navigate back to Table 1.

## Table 2 Long description

A table comparing physical characteristics of treatment and control groups. The table has 6 rows and 5 columns. The columns are labeled Characteristics, Treatment, Control, and p-Value. The rows are labeled Age, years, Height, cm, Body mass, kg, Body fat, percent, and Aerobic exercise per week, minutes. The treatment group has 60 participants (18 males) and the control group has 56 participants (19 males). Row 1: Age, years, Treatment: 21 ± 3, Control: 20 ± 1, p-Value: 0.4083. Row 2: Height, cm, Treatment: 169 ± 11, Control: 171 ± 10, p-Value: 0.4155. Row 3: Body mass, kg, Treatment: 69 ± 16, Control: 75 ± 29, p-Value: 0.2021. Row 4: Body fat, percent, Treatment: 23 ± 9, Control: 23 ± 9, p-Value: 0.7457. Row 5: Aerobic exercise per week, minutes, Treatment: 239 ± 268, Control: 198 ± 244, p-Value: 0.4024.

Navigate back to Table 2.

## Table 3 Long description

A table comparing performance metrics between treatment and control groups. The table has 10 rows and 6 columns. The columns are labeled Characteristics, Treatment N = 60 (18 males), p-Value, Control N = 56 (19 males), and p-Value. The rows are labeled PVT pre (msec), PVT post (msec), Delta, MemTrax % correct pre, MemTrax % correct post, Delta, MemTrax RT pre (sec), MemTrax RT post (sec), Delta, Handgrip pre (kg), Handgrip post (kg), and Delta. The table presents values for each characteristic under the treatment and control groups, along with corresponding p-values. Notable trends include improvements in PVT post times and MemTrax RT post times for both groups, with significant p-values indicating statistical significance in some cases.

Navigate back to Table 3.
